# Does age-dynamic movement accelerate facial age impression? Perception of age from facial movement: Studies of Japanese women

**DOI:** 10.1371/journal.pone.0255570

**Published:** 2021-08-05

**Authors:** Motonori Kurosumi, Koji Mizukoshi, Maya Hongo, Miyuki G. Kamachi

**Affiliations:** 1 Graduate School of Informatics, Kogakuin University, Shinjuku, Tokyo, Japan; 2 POLA Chemical Industries, Inc., Tokyo, Japan; 3 Faculty of Informatics, Kogakuin University, Shinjuku, Tokyo, Japan; Institut VEDECOM, FRANCE

## Abstract

We form impressions of others by observing their constant and dynamically-shifting facial expressions during conversation and other daily life activities. However, conventional aging research has mainly considered the changing characteristics of the skin, such as wrinkles and age-spots, within very limited states of static faces. In order to elucidate the range of aging impressions that we make in daily life, it is necessary to consider the effects of facial movement. This study investigated the effects of facial movement on age impressions. An age perception test using Japanese women as face models was employed to verify the effects of the models’ age-dependent facial movements on age impression in 112 participants (all women, aged 20–49 years) as observers. Further, the observers’ gaze was analyzed to identify the facial areas of interests during age perception. The results showed that cheek movement affects age impressions, and that the impressions increase depending on the model’s age. These findings will facilitate the development of new means of provoking a more youthful impression by approaching anti-aging from a different viewpoint of facial movement.

## Introduction

An individual’s face is an information source communicating knowledge about their individual characteristics such as gender and age, as well as their physical and mental conditions, such as state of health and emotions [1,2]. In particular, facial youthfulness is emerging as a major motivating factor in the coming super aging society [3]. Moreover, among Caucasians, age impression from the face has been proposed as a biomarker of aging [4,5], and looking older than one’s actual age is associated with increased mortality [4,6]. Thus, the importance of facial youthfulness goes beyond a high sense of beauty and is relevant to health and well-being.

Previous studies on age impression based on facial shape [7,8] and skin appearance [9–16] have been reported. While the facial shape cannot be easily changed, the skin appearance can be controlled by cosmetics. As a result, people (especially women) are very interested in their own skin condition. Previous studies have reported mainly on static skin conditions by investigating the effects of morphological and tonal characteristics, such as wrinkles, sagging, spots, and unevenness of color [9–16]. However, the facial skin we interact with in real life is constantly moving during conversation, and we perceive various impressions from moving faces. In fact, we may have the experience of feeling both youthful and old at fleeting moments in our conversations. Therefore, in order to elucidate the overall impression, in real life, it is necessary to consider the effects of skin movement.

Conversely, there have been some reports focusing on the facial motion information in person identification and recognition of facial expressions. Regarding the effect of motion information on person identification, it was found that the correct response rate was higher for video images than for still images [17,18], and that people can discriminate both between individuals and between males and females from motion-based information alone [19]. In terms of the effect on recognition of facial expressions, the speed at which they change contributes to the perception of emotions [20], and facial motion information increases the correct response rate for facial expression recognition [21]. Thus, facial motion information is an important clue for face and facial expression recognition. However, there are few reports regarding the effects of facial and skin movement on age impression. Previous studies have focused on the motility of muscles, such as facial and skeletal muscles. Nevertheless, what we see in others’ facial expressions are not the muscles themselves but the soft skin tissues covering the muscles. These soft skin tissues are known to lose viscoelasticity with age [22]. Therefore, the motility of the skin surface when adopting an expression must change with age. One may wonder how a slight difference in skin movement can affect the overall impression of the face. It is known that human face recognition is mediated by the face-specific cortical areas of the brain, and its abilities become proficient with experience [23,24], enabling humans to detect even slight differences, such as the placement of facial parts (e.g., the distance between the eyes and mouth). Thus, it can be assumed that we perceive age from slight differences in skin movement. Based on the assumption that aging causes a decline in skin movement, we hypothesized that facial movement inherently creates a lively and youthful impression on others, but age-related deterioration of skin movement may dilute the youthful effect of that impression.

## Materials and methods

In this study, we performed an experiment comprising two investigations. An age perception test using Japanese women as face models and observers was employed to verify: (1) the facial areas of interest focused on during age perception by the observers’ gaze analysis, and (2) the effects of the models’ age-dependent facial movements on age impression. As we observe faces from various angles in daily life, facial stimuli in this study were also recorded and presented from four observation angles. The main objective of this study was to examine facial skin dynamics and observer’s gaze during age judgment. In addition, we briefly report the effect of the angles on the models’ age impression, wherein a pronounced movement effect was identified.

### Ethics statement

All demonstration tests related to this study were performed according to the protocol approved by the ethical committee of POLA Chemical Industries, Co., Ltd., in accordance with the Declaration of Helsinki (Approval No. 2015-G-005; January 16, 2015). Written informed consent has been given (as outlined in the PLOS ONE consent form) to publish these case details. Two participants used as images in the figures has given informed consent including the permission to use the facial images in an online open-access publication.

### Participants

A total of 112 Japanese women aged 20–49 years participated as observers who evaluated age impression. It was reported that, as a decline in visual perceptual function, a decline in the ability to adapt to light and dark is gradually observed from the 40s, and becomes more pronounced over 50s [25]. In addition, it was reported that as a result of evaluating the reaction time to memorized cards for each age group in terms of the cognitive processing speed of perceived information, it was confirmed that the reaction time was longer in the 50s than in other younger groups [26]. For these reasons, in this study, the exclusion criteria for the subjects in the previous study was set to be in their 50s or later. Table 1 shows the age breakdown of the observers.

**Table 1:** Participant characteristics.

Age group (years)	N	Mean	S.D.
Participants (Observer)			
20–29	37	24.97	2.92
30–39	38	34.68	2.82
40–49	37	43.89	3.30
Stimuli (Face model)			
20–29	16	25.38	2.90
30–39	16	35.19	2.79
40–49	16	44.50	3.08
50–59	16	54.50	3.27
60–69	16	62.88	2.36

### Stimuli

The facial models were 80 Japanese women aged 20–69 years (divided into five age groups with 10-year increments, with 16 facial models in each age group). Table 1 shows the breakdown by age. All models were unfamiliar to the observer. In order to eliminate the influence of individual differences in make-up, the models were asked to participate in this experiment without make-up.

**Fig 1:**
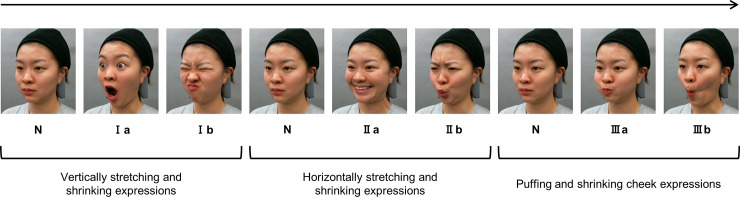
Facial expression sequence used as stimuli. Photograph N shows neutral facial expression; Ia, lowering chin with opening mouth expression; Ib, vertically shrunk facial expression; IIa, horizontally opening mouth expression; IIb, horizontally closing mouth expression; IIIa, puffing cheek expression; IIIb, shrinking cheek expression. The roman numerals I, II, and III indicate vertically stretching and shrinking mouth expressions, horizontally stretching and shrinking mouth expressions, and puffing and shrinking cheek expressions, respectively. The facial expressions changed every second. The long arrow over the facial photographs shows the stream of time.

Each model expressed facial movements from neutral face (N) to lowering chin with open mouth expression (Ia); vertically shrunk face expression (Ib); from neutral face (N) to horizontally opened mouth expression (IIa); horizontally closing mouth expression (IIb); and from neutral face (N) to puffing cheek expression (IIIa) to shrinking cheek expression (IIIb) so that the expression switches every second using a metronome set at 60 beats per minute (bpm) (Fig 1). Each expression was taught to the models based on the action unit defined by the Facial Action Coding System proposed by Ekman et al. [27]. Since the intensity of facial expression varied between models, the effects to perceived age were compared within the exercise condition.

**Fig 2:**
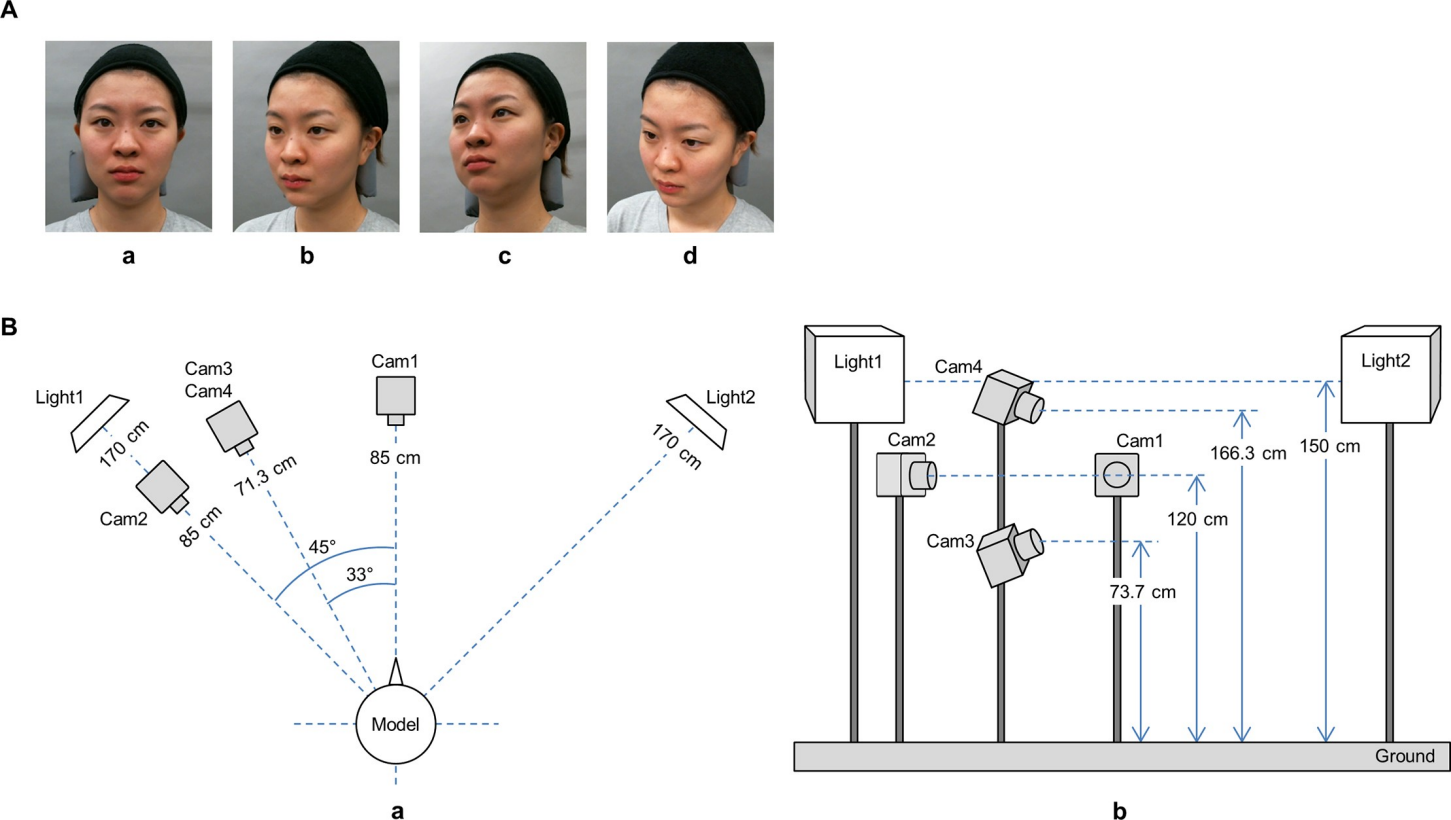
(A) Viewpoint used as stimuli. Dynamically-changing facial expressions were filmed with a digital video camera from four different directions: (a) 0° rotation (front), (b) 45° rotated right in the yaw (right side), (c) 33° rotated right in the yaw and pitch above (right upward), and (d) 33° rotated right in the yaw and pitch below (right downward). (B) Equipment layout to film with a digital video camera. Dynamically changing facial expressions were filmed with a digital video camera from four different directions: (a) upper view of equipment layout, (b) front view of equipment layout.

As the area of the face skin changes depending on the observation angle, the effect of movement may differ depending on the observation angle. In this study, the face was photographed at four angles: (a) front, (b) 45° rotated right in the yaw, (c) 33° rotated right in the yaw and pitch below, and (d) 33° rotated right in the yaw and pitch above (Fig 2A). These four angles were selected as the facial observation angles that we come across in daily life. In addition to the frontal direction, we used the horizontal direction and the up and down oblique direction, which is a compound of the rotation axis, considering the application to other angles. Note that in order to focus on the relationship between facial dynamics and age, changes in the recorded factor of observation angles were ignored intentionally in the analysis.

A video of the face model was recorded with a camera (GV90C, Library, Japan) installed at the four angles described above. Fig 2B shows the installation conditions of the cameras and lights.

**Fig 3:**
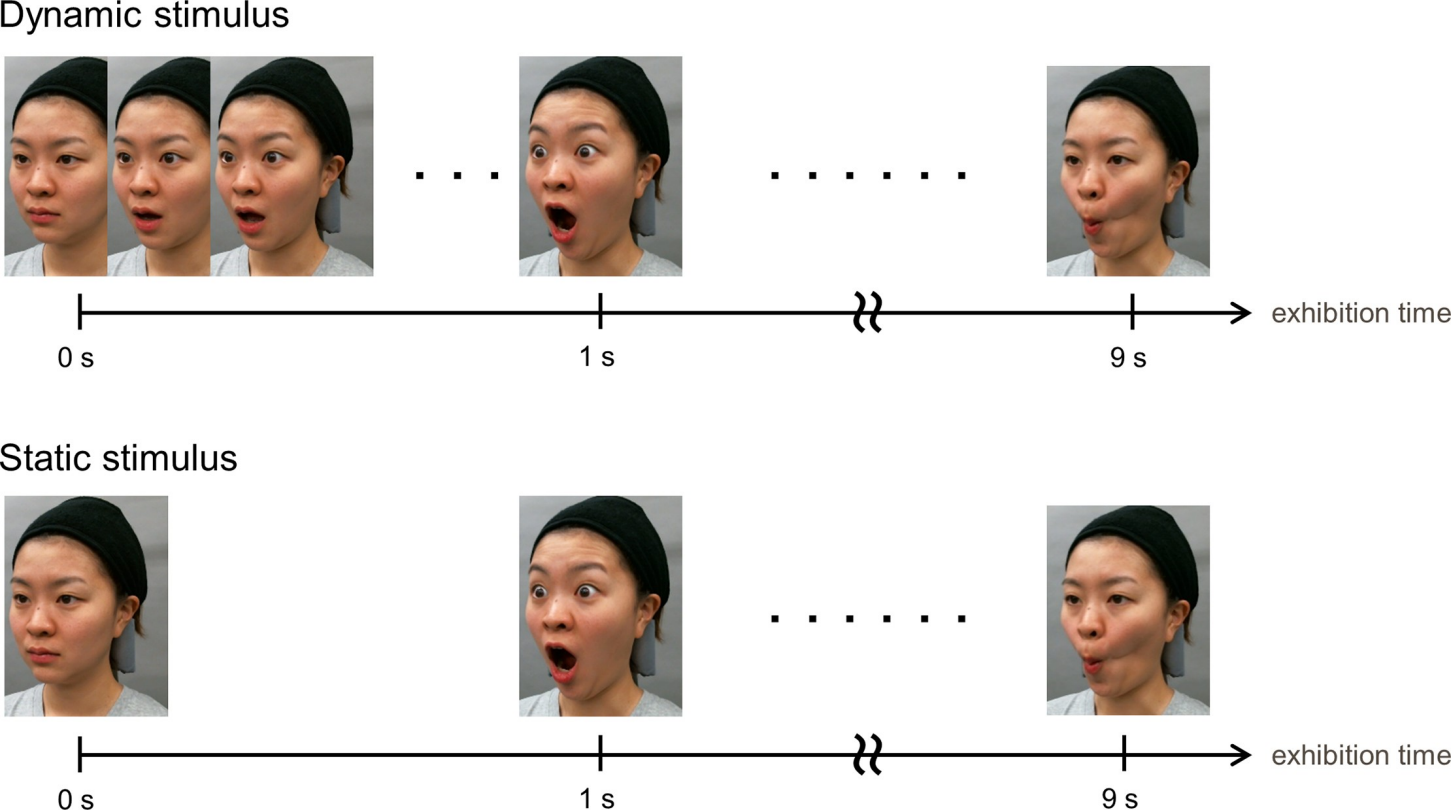
Two types of stimulus-conditions. Photographs show two stimulus-conditions: Dynamically moving expression shown by a nine-second video clip at a speed of 30 frames per second (fps) containing nine facial expression processes as the “dynamic stimulus,” and static facial expressions shown by nine static images taken from video files at the highest degree of facial expression, with a speed of 1 fps as the “static stimulus”.

Two kinds of stimuli were produced as follows: A movie for a total of nine seconds in which the moving images of N-Ia-Ib, N-IIa-IIb, and N-IIIa-IIIb were linked. These were defined as “dynamic stimulus.” Another nine-second video linked the nine still images at the moments when the intensity of each facial expression was the highest. This was the “static stimulus” (Fig 3). The frames with the maximum expression intensity were defined using the frontal video as follows: the frame with the maximum or minimum distance between trichion and gnathion for vertical movements; the frame with the maximum or minimum distance between the left and right cheilions for horizontal movements; and the frame in which the area occupied by the face was the largest or smallest for puffing and shrinking cheek movements. The face size was set as what it appears to be at a distance at which humans talk with each other on a daily basis. The face width (horizontal distance between the zygomatic arches) was 12 cm (11.421 degree of visual angle) for each face model on a 24-inch color liquid crystal display (Color Edge CX2414, EIZO, Japan). The stimulus size was 700 pixels horizontally and 800 pixels vertically. The face stimuli were observed at a viewing distance of 60 cm.

The following two points were considered in creating visual stimuli to confirm the effects of skin movement only on age impression, excluding other characteristics of faces. At first, since facial expressions had a substantial impact on bias of age estimation[28], in this study, facial movements are expressed as simple, independent movements, such as vertical and horizontal stretching and shrinking mouth expressions, and puffing and shrinking cheek expressions. In addition, static facial expressions appeared as a “static stimulus,” consisting of nine static images (1 fps each) taken from video clips, with each facial expression at its highest intensity. The effect of static facial expressions themselves was thus eliminated under the consideration of its combination effect.

The second point is that the decrease in the motor function of the facial muscles, not skin dynamics, may inhibit skin movement as the model creates facial expressions. Therefore, it was necessary to employ a way to exclude the overall duration of movement as a factor. In our study, facial movement was temporally controlled during recording in accordance with the metronome, so the prescribed facial movements could be expressed at the correct time. The models were trained to vary their facial expressions before the experiment. Furthermore, one facial model whose intensity and timing of expression deviated from the experimenter’s designation was excluded in advance.

### Specification of the facial location for age estimation by analyzing gaze data

**Fig 4:**
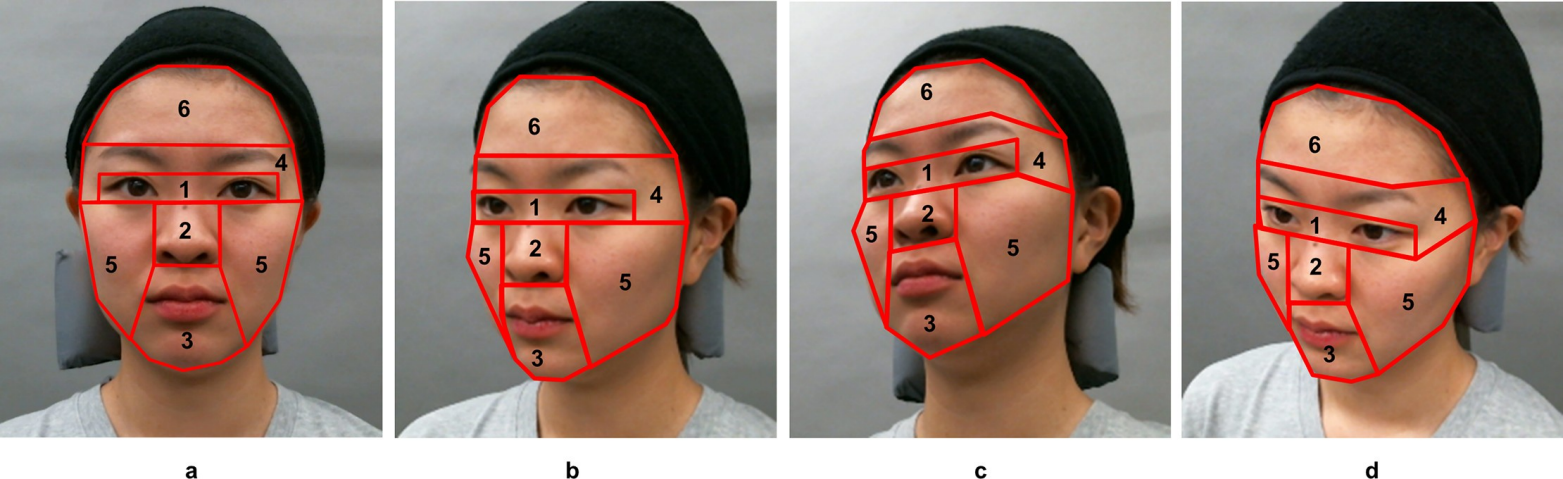
The regions of interest used for specifying the location for evaluating age. To specify the facial location used for evaluating age, six regions of interest (ROI) were selected: Eyes, nose, mouth, and the area surrounding the eyes, that is, eyebrows, cheek, and forehead in four directions: (a) 0° rotation (front), (b) 45° rotated right in the yaw (right side), (c) 33° rotated right in the yaw and pitch above (right upward), and (d) 33° rotated right in the yaw and pitch below (right downward).

To specify the facial location where observers unconsciously estimate the age of others, the gazes of the 112 observers described above were recorded with an eye-tracking system (Tobii Pro X2-30) (Tobii, Stockholm, Sweden). Sampling rate of the eye tracking system was 30 Hz and a fixation was determined by means of a velocity-based filter (Tobii I-VT filter [29], velocity threshold 30 degrees/second and minimum fixation duration of 67 ms). The following six regions of interest (ROI) were selected: eyes, nose, mouth, surrounding area of the eyes, including brows, cheeks, and forehead (Fig 4). Ratios of gaze-fixing time for the six ROIs in the faces were calculated during the estimation of age impression. The two types of stimuli (dynamic and static) and observation angles of the face were integrated and analyzed.

### Age perception test

**Fig 5:**
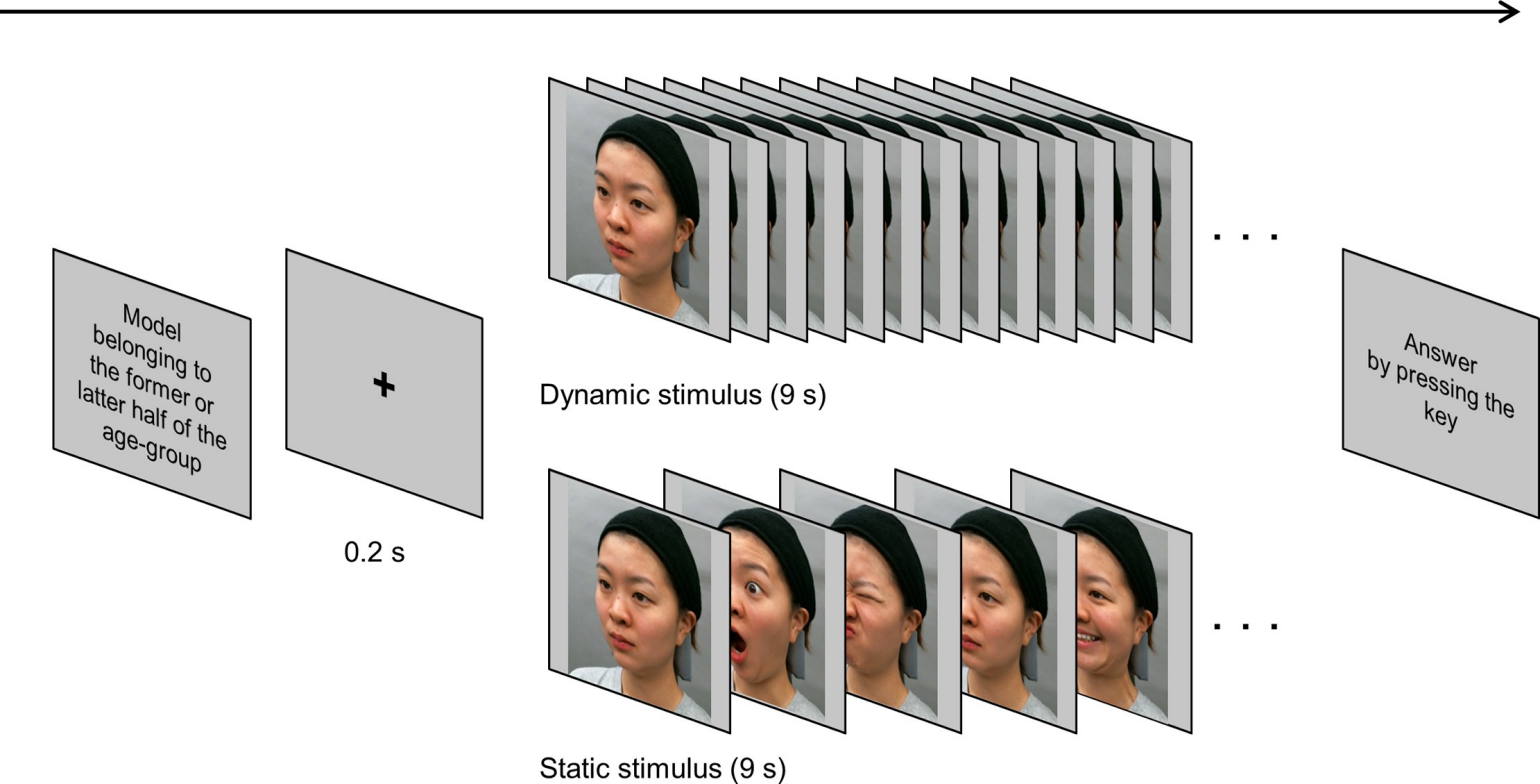
An example of the procedure of the age-cognitive experiment. After looking at the introduction part of the movie, the observers were asked to fix their gazes at a center point on a liquid-crystal display (LCD) for 0.2 s, observe the dynamic stimulus or static stimulus for 9 s, and answer to which half of the age group, the former or latter half, the model belonged by pressing a button.

First, the observers were told the age group that the models belonged to, and then they were allowed to observe dynamic stimulation or static stimulation for 9 s. They were assigned a two-alternative forced choice task to answer which half of the age group—former or latter—the model belonged to by pressing the appropriate keys (Fig 5). The observer watched the faces of all 80 models as they appeared on a display, once for each model, and evaluated the age impressions. The models’ video images as watched by the observer were randomly selected from eight different facial-expression video files. There were four moving and four static facial images taken from four different directions. The order in which each image was presented was random, and the number of presented stimuli was counterbalanced by facial models and viewing angles.

To evaluate the effects of the models’ facial movements on their age impressions, the percentage of the models belonging to the latter half of the age group was calculated; a two-way analysis of variance (ANOVA) with dynamic and static faces, and the five different age groups was conducted; and multiple comparison test with Bonferroni correction was performed. We also examined the factors associated with the observation angle in models whose facial movements significantly influenced the age impression. All statistical analyses were performed using SPSS, version 24.0 (IBM, Armonk, NY, USA).

## Results

### Specification of the facial location for age estimation by analyzing gaze data

**Fig 6:**
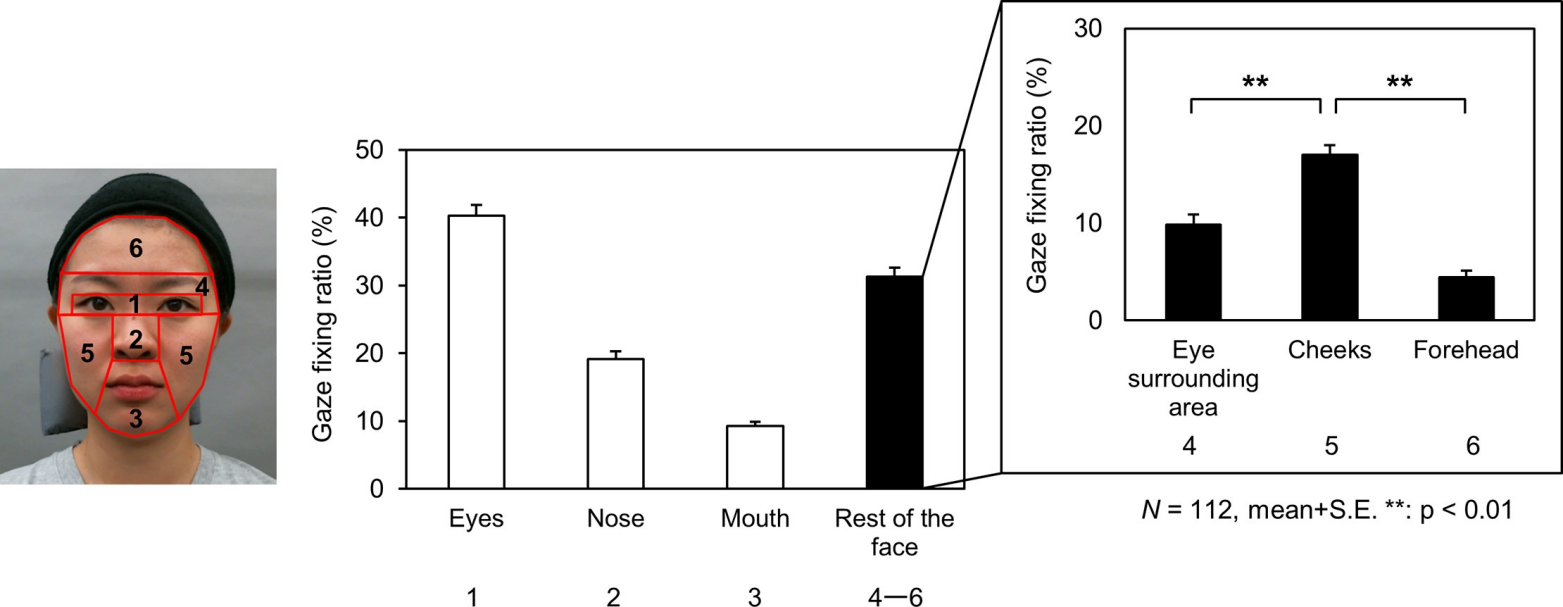
Specific locations on the face for evaluating age by analyzing gaze data. The percentages of gaze-fixing time for the six regions of interest (ROIs) were calculated during the estimation of age impression. The closed and open columns indicate the percentages of gaze-fixing times for the six ROIs.

The percentages of gaze-fixing times for six ROIs where the observers evaluated the age of the models are depicted in Fig 6. The observation or angles of the face were integrated and analyzed. In summary, during the estimation of the ages, the results were confirmed that the gazes were focused not only on the eyes, nose, and mouth but also on the facial skin area, especially on the cheeks [*F* (1, 111) = 523.567, *p* < .001, *η*^*2*^ = 0.825].

### Age perception test

**Fig 7:**
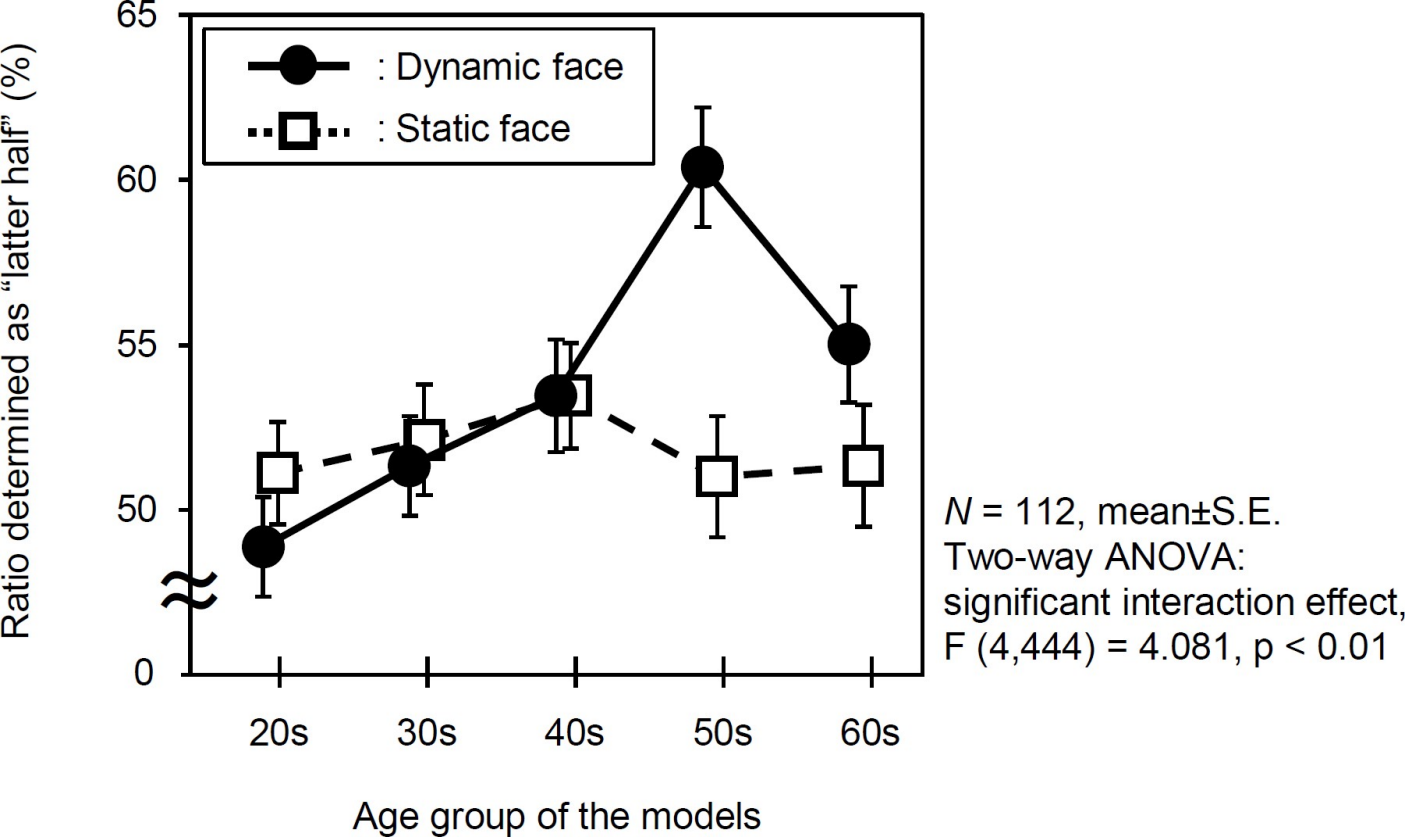
Effects of facial movements on age impression. The graph shows the percentages of 112 observers who thought the models to be in the older group, and their impressions after the dynamic and static conditions. The closed circles (●) and open squares (□) show the percentages of observers stimulated by dynamic and static stimulations, respectively. The marks and the vertical lines on the marks are mean values and standard errors, respectively.

Our assessment confirmed that the effects of skin movement on age judgment differ depending on the age of the face model, and that skin movement increases the age impression as the age of the model increases. Specifically, the two-way ANOVA with models’ age groups (five: in their 20s, 30s, 40s, 50s, and 60s) and facial movements (two: dynamic and static) as independent variables on perceived age (the estimated ratio of “the latter half” of the age group; 25–29 years old in the 20s group) was performed. The result showed a significant interaction between age groups and facial movements [*F* (4, 444) = 4.081, *p* = 0.003, *η*_*p*_^*2*^ = 0.035]. A simple main effect of movement was confirmed, indicating that only models in their 50s experienced a strong effect of dynamics [*F* (1,444) = 11.753, *p* = 0.001, *η*_*p*_^*2*^ = 0.096]. The 60s group was considerably higher in the dynamic condition, though the difference was not statistically significant [*F* (1,444) = 2.224, *p* = 0.139, *η*_*p*_^*2*^ = 0.020] (Fig 7).

**Fig 8:**
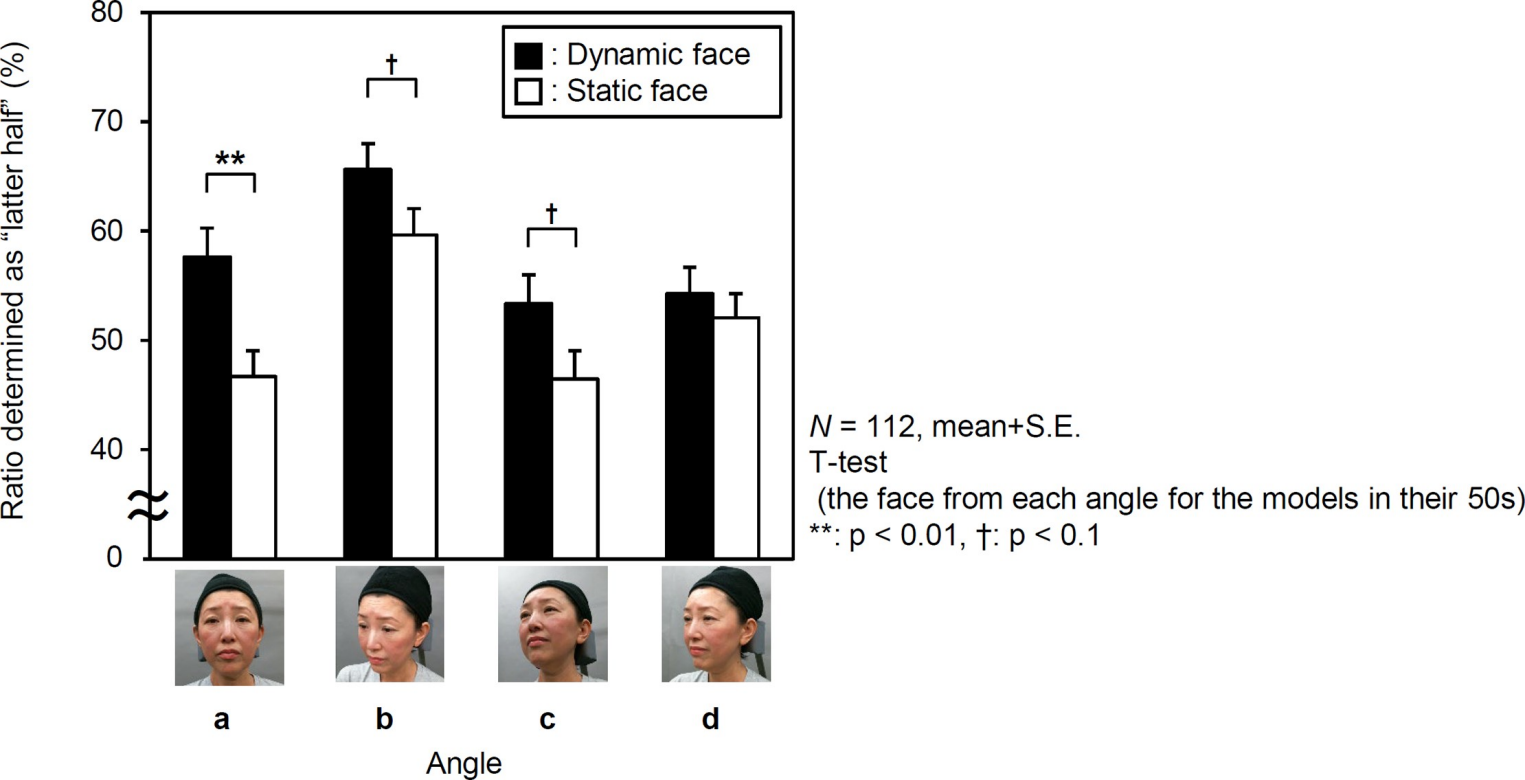
Viewpoint depended on the effects of facial movement on age impression. The graph shows the percentages of 112 observers who perceived the models in their 50s to be in the latter half of this decade based on the models’ age-impressions using the dynamic and static cognitive stimulations as a function of four directions: (a) 0° rotation (front), (b) 45° rotated right in the yaw (right side), (c) 33° rotated right in the yaw and pitch above (right upward), and (d) 33° rotated right in the yaw and pitch below (right downward). The closed columns (■) and open columns (□) show the percentages of observers stimulated by dynamic and static stimulations, respectively. The marks and vertical lines on the marks signify mean values and standard errors, respectively.

As faces are observed from various angles in our daily life, facial stimuli were also recorded and presented from various viewing angles. The effects of facial movement on the impression of being perceived as older diminished in some specific viewing angles. Specifically, the more the face orientation increased in the lateral (yaw) direction, the weaker the effects of the dynamics became. This tendency was especially confirmed with face models in their 50s (Fig 8). As a result, only in the frontal view (the angle of the yaw direction being zero), facial movement shows a significant increase in age impression [*t* (111) = 3.040, *p* = 0.003, *d* = 0.431]. When observed from the upper right and lower right observation angles (both angles in the yaw directions at 33 degrees), marginal effects were found; upper right [*t* (111) = 1.927, *p* = 0.056, *d* = 0.243], lower right [*t* (111) = 1.817, *p* = 0.072, *d* = 0.259]. Moreover, at the observation angle of 45 degrees to the right (yaw direction), there was no effect of facial movement [*t* (111) = 0.673, *p* = 0.503, *d* = 0.080].

## Discussion

Previous studies have mainly focused on static faces, reporting that aging characteristics of the skin, such as wrinkles, spots, and sagging, affect age impression [9–16]. In this study, we examined the effects of facial movement on age impression, based on the hypothesis that dynamic skin condition has a significant influence on judged age impression. In order to identify face areas considered important for age judgment, the observer’s gaze was also measured during trials. The results clearly suggested that the cheek skin is frequently observed when humans estimate a person’s age. The results demonstrated that facial movement affects age impressions, and that the impressions increased depending on the person’s age. This confirmed that in the facial models aged 50 years, the person tends to be regarded as older when the face moves.

The gazes of all participants were recorded to specify the facial regions where observers subconsciously look for estimating the age of others. Previous studies have reported that with a few racial differences, human gazes are concentrated on facial features such as the eyes, nose, and mouth for identifying others [30–33]. The present findings showed that when humans judge the ages of others, they observe not only areas with high visual prominence, such as the eyes, nose, and mouth, but also areas including skin, especially cheeks.

Previous studies have reported that human gaze behavior is affected not only by the saliency of visual information (bottom-up processing) but also the exploratory tasks during observation (top-down processing) [34]. When people identify others by their faces, although this may vary due to cultural differences, the gaze typically concentrates on the eyes, noses, and mouths of the observed persons. In adult faces, the relative arrangement and individual morphology of the facial structure hardly changes after the growth of the skeleton. Therefore, the eyes, nose, and mouth provide important information for identifying an individual. Conversely, when judging the age of an adult face, skin with more pronounced aging changes, especially the cheek area with laugh lines and sagging skin, and the area surrounding the eyes that is constantly moving, may be a useful source of age-related information. This study found that the observers’ gaze was concentrated on the cheeks when judging the age of others, indicating that humans empirically use the skin for judging age.

The results of age impressions under dynamic/static conditions, with five different age groups revealed that there was a significant interaction. While younger models in their 20s to 40s had facial movements that did not give an older impression to others, the facial movements of models in their 50s gave others an older impression. This is speculation on the part of the authors, but it is likely that as the model gets older, facial movements will act in the direction of increasing perceived age. We hypothesized that age-related changes in skin movement would attenuate the active, youthful impression of the face. However, in the elderly models, rather than attenuating a youthful impression, the movement of the skin makes the face look older.

Our experiments suggested that skin movements changes skin appearance and affects age impression. We discuss that this happens mainly due to the following two reasons.

First, changes in the skin’s physical properties may reduce the motility of the skin surface. The movement of the facial skin surface is caused by deeper layers of active facial muscle, as radiated through the inside of the subcutaneous tissue and skin. Structures involved in viscoelasticity, such as collagen, elastin, and dermal papilla, run throughout, and are known to undergo qualitative and quantitative changes with aging [35–38]. Therefore, with age, changes in the physical properties of the subcutaneous tissue and skin affect the smooth motility of the skin surface. It has been reported that humans can perceive the activity of the subcutaneous tissue simply from the motility of points on the body surface as “biological motion” [39]. In this study, facial movement caused by skin around the cheek, not the edge around the features such as eyes and mouth, as surface information increased age impression in facial models aged in their 50s. This result suggests that when making an age judgment, humans perceive sensitively subtle changes in the physical properties of the skin that decrease the motility of the skin surface. Human perceivers expect smooth movement of an expressive face. Younger people in static pictures are recognized almost in the same manner as with dynamic moving faces. However, elder skin is probably detected through rough and unsmooth movement and arise the sense of discomfort of the motion.

Second, the cheek skin movement may expose a static aged appearance. In general, wrinkles that occur as a sign of aging are confirmed on a static face by plastic deformation due to degeneration of elastic fibers. However, when we consider facial expressions, temporary wrinkles due to elastic deformation also occur by the contraction of the skin surface. The appearance of temporary wrinkles due to this elastic deformation becomes more frequent due to changes in the physical properties of the skin [40]. This study confirmed that facial movement increases age impression in elderly models. The prominent temporary wrinkles in the aged skin are considered to cause the aged impression.

The results of the present study partly show that there are some reasons, which cannot be explained only by dynamics. For example, the effect of movement on age impression peaks in the 50s and weakens in the 60s. It is known that wrinkles and sagging suddenly become apparent both histologically and as appearance changes in women in their 60s [22,35,41,42]. Therefore, it is considered that aging characteristics that appear in a static state, such as wrinkles and sagging, attenuate the apparent aging effect of movement in one’s 60s.

We also examined the factors associated with the observation angle in models in their 50s whose facial movements increased the age impression. The effect of movement on age impression was significant for the frontal face, but decreased as the face was inclined in the lateral (yaw) direction. Previous studies have reported that the symmetry of facial movement affects age impression [43]. This indicates that the effect of the movement on age impression was maximized in the frontal face, as the lateral symmetry of facial movement was easily detected.

This study has some limitations. First, in order to verify the effect of facial movements on age impression, the models in our experiment were asked to make simple facial movements, such as expansion and contraction, swelling, shrinking, and exercises with high expression intensity. However, it is worth considering that actual quotidian conditions, such as smiles and facial expressions when speaking, may vary from the expressions used in this experiment. Next, to prioritize the effect of facial movement on age impressions, the effect of the face observation angle was mentioned only for models in their 50s who had significant older impressions in the dynamic condition. In the future, the relationship between facial movement and observation angle should be examined in more detail for more suitable and real-life conditions. Furthermore, this study was conducted with Japanese women, and in order to eliminate the influence of individual differences in make-up, the models were asked to participate in this experiment without make-up. The observers were limited to females because it was assumed that men and women would have different opportunities to come into contact with female faces with no make-up, which would have some effect on perceived age. Further study is needed to determine whether the present results are similar for different races, sexes, and make-up faces. Finally, we discussed the effects on age impressions based on the hypothesis that cheek skin movement and its appearance change with age. In the future, the changes in cheek skin movement and appearance with aging need to be clarified metrologically.

## Conclusions

In this study, we examined the effect of facial movement on age impression and drew the following conclusions based on the findings. Women in their 50s may give an age impression while talking to others. This age impression is a result of the dynamic features of the cheek skin. Many of us may only recognize our own static faces in mirrors and photographs on a daily basis. However, this study elucidates the importance of targeting dynamic faces based on which others make age impressions. In the future, if we can clarify the characteristics of the skin’s physical properties that cause differences in skin appearance due to facial movements, we will be able to provide measures to maintain a younger impression on a daily basis.
